# Estimating the global burden of diarrhea among young children with wasting and stunting and its projection to 2050: evidence from the Global Burden of Disease 2021 study

**DOI:** 10.3389/fped.2026.1871122

**Published:** 2026-07-01

**Authors:** Lingling Dai, Zhenni Fan, Yijia Fan, Mingfang Ping

**Affiliations:** Department of Pediatrics, The Second Hospital Affiliated to Jiaxing University, Jiaxing, Zhejiang Province, China

**Keywords:** childhood diarrhea, Global Burden of Disease, projection, stunting, temporal trend, wasting

## Abstract

**Background:**

Diarrheal disease is one of the leading causes of death among children worldwide. Childhood stunting and wasting are independent risk factors for pediatric diarrhea. Using data from the Global Burden of Disease 2021 study (GBD 2021), this study investigated the epidemiological burden, temporal trends and future trajectories of stunting- and wasting-related diarrhea in children under 5 years globally, so as to provide evidence for formulating targeted and differentiated diarrhea prevention and control strategies.

**Methods:**

This study adopted GBD 2021 data from 1990 to 2021 covering 204 countries and regions worldwide. Four core indicators including deaths, years of life lost (YLLs), years lived with disability (YLDs) and disability-adjusted life years (DALYs) were used to compare the burden of the two types of diarrhea. Stratified analyses were performed by sex, age, region and Socio-Demographic Index (SDI). Estimated annual percentage change (EAPC) was calculated to quantify temporal trends, and decomposition analysis was conducted to estimate the contributions of epidemiological changes and population growth to burden fluctuations. The Autoregressive Integrated Moving Average (ARIMA) model was applied to predict and compare long-term burden trends from 2022 to 2050. As a retrospective secondary study, it has limitations due to raw data quality, regional data imbalance and model estimation bias, and the predictions only reflect overall trends.

**Results:**

From 1990 to 2021, all burden indicators of stunting- and wasting-related diarrhea in under-five children declined substantially and continuously, with Western Sub-Saharan Africa bearing the highest burden globally. Projections for 2022–2050 showed sustained reductions in mortality and disability-adjusted life years, with a more remarkable decline among girls. Wasting-related diarrhea experienced a greater and faster burden reduction, and obvious heterogeneity existed in their long-term trends.

**Discussion:**

The steady decline in disease burden is mainly attributed to improved epidemiological conditions. Improving sanitation facilities, ensuring access to safe drinking water, optimizing child nutrition and increasing rotavirus vaccination coverage are core measures for diarrhea control. ARIMA projections revealed that wasting-related diarrhea saw a far larger burden reduction. Early and precise nutritional interventions for wasted children can effectively reduce the burden of acute diarrhea and achieve efficient and sustainable prevention and control.

## Introduction

The World Health Organization defines diarrhea as an intestinal condition characterized by three or more episodes of loose or watery stools within a 24-h period. It arises from excessive intestinal fluid secretion or impaired epithelial electrolyte transport, leading to disturbances in water, sodium, and potassium balance. Severe diarrhea may cause dehydration and metabolic disorders, posing a serious threat to early childhood health (1–3). As a critical global public health challenge, diarrhea ranks as the third leading cause of death across all age groups and the second leading cause of mortality in children under 5 years worldwide (4–7). In 2023, there were 1.7 billion new global diarrhea cases, resulting in over 1.8 million annual deaths. Children under five are highly susceptible, with a global prevalence of 24.9%, accounting for 18% of all diarrhea-related deaths globally, particularly in developing countries such as Indonesia (8). Pediatric diarrhea mortality is disproportionately concentrated in low-income regions, with contribution rates of 12% in Africa and the Eastern Mediterranean and 11% in Southeast Asia, reflecting substantial global health disparities (9). The etiology of childhood diarrhea is multifactorial. In addition to bacterial enteric infections, major modifiable risk factors include poor environmental sanitation, unsafe drinking water, low rotavirus vaccine coverage, inadequate oral rehydration salt utilization, zinc and vitamin A deficiency, stunting, underweight, and wasting (10–15). Benefiting from improvements in public health infrastructure, water and environmental sanitation, pediatric nutritional care, and standardized rehydration therapy, the global incidence and mortality of childhood diarrhea have gradually declined over the past three decades (16).

The interactive detrimental relationship between malnutrition and childhood diarrhea remains a key research priority in child health (17). Notably, stunting and wasting demonstrate a specific bidirectional vicious cycle with diarrhea, critically influencing diarrhea onset, persistence, and clinical prognosis (18). Accumulating epidemiological evidence confirms that stunting and wasting serve as independent risk factors for childhood diarrhea. Malnutrition impairs intestinal mucosal integrity and immune defense, increasing susceptibility to enteric pathogen invasion and elevating the risks of diarrhea occurrence and severe progression (10, 17, 18). Conversely, recurrent or persistent diarrhea depletes nutritional reserves, disrupts intestinal absorption and metabolism, hinders physical and neurological development, and further exacerbates malnutrition, forming a self-perpetuating cycle between malnutrition and diarrhea (1, 19, 20). The combined adverse effects of persistent diarrhea and malnutrition impede childhood growth and immune maturation, cause irreversible brain developmental damage, increase the risk of recurrent illness in later life, and substantially contribute to disability-adjusted life year loss in children worldwide (2, 18). Existing studies mainly focus on individual risk factors, regional epidemiological characteristics, or generalized nutrition-diarrhea associations. However, few studies have systematically differentiated stunting from wasting, and large-scale refined comparative analyses regarding their global spatiotemporal distribution, long-term trends, driving mechanisms, and future projections remain limited. The similarities and heterogeneity in pathogenic features and intervention sensitivity between the two malnutrition subtypes have not been fully elucidated.

To address these research gaps, this study utilized longitudinal data from the Global Burden of Diseases, Injuries, and Risk Factors Study 2021 (GBD 2021). The study population included children under 5 years of age across 204 countries and territories from 1990 to 2021. This study systematically evaluated the global burden and temporal trends of diarrhea attributable to stunting and wasting. Multidimensional stratified analyses were performed by Socio-demographic Index, sex, and age to compare epidemiological disparities and dynamic patterns between the two types of nutrition-related diarrhea. Trend quantification and driver decomposition analyses were conducted to clarify the regulatory effects of epidemiological improvements and population growth on disease burden. The autoregressive integrated moving average (ARIMA) model was further employed to predict long-term burden trends from 2022 to 2050. This study highlights the epidemiological commonalities and heterogeneity of diarrhea associated with stunting and wasting and clarifies their distinct intervention implications. The findings provide refined evidence for developing hierarchical and precise global childhood diarrhea prevention and control strategies, optimizing regional health resource allocation, breaking the malnutrition-diarrhea vicious cycle, and reducing the global morbidity and mortality burden of pediatric diarrhea.

## Methods

### Data source

In this study, data on diarrheal burden attributable to stunting and wasting were derived from the Global Burden of Disease (GBD) Study (http://ghdx.healthdata.org/gbd-resultstool) without involvement of human subjects. The GBD study established a publicly available database containing standardized estimates of attributable burden for multiple risk factors across all countries (21). This study employed four officially recognized metrics of the Global Burden of Disease (GBD) Study, including number of deaths, years of life lost, years lived with disability and disability-adjusted life years, as fundamental evaluation indicators. Several comprehensive assessment frameworks and attribution criteria were developed independently by the research team.

The Sociodemographic Index (SDI) serves as a metric for assessing regional development levels, which are strongly correlated with population health outcomes. Its value derives from the geometric mean of three normalized components, each ranging from 0 to 1: the fertility rate among women younger than 25 years, the mean years of schooling for individuals aged 15 years or older, and lag-distributed income per capita. For the GBD 2021 study, the computed SDI values were rescaled by a factor of 100 to enhance interpretability. In this framework, a score of 0 denotes the lowest possible level of health-related development, while a score of 100 signifies the highest. Methodological details regarding the construction of the SDI are elaborated in a key GBD 2021 publication, which also classifies 204 countries and territories into five SDI categories—low, low-middle, middle, high-middle, and high—according to their 2021 estimates (22).

### Risk factors

Key contributors to diarrheal disease risk include inadequate hand hygiene, suboptimal rotavirus immunization rates, lack of access to safe sanitation facilities, consumption of contaminated drinking water, insufficient zinc intake, child growth faltering (including stunting, underweight, and wasting), infrequent use of oral rehydration salts, adverse birth outcomes such as low birthweight and preterm delivery, absence or early cessation of breastfeeding, and inadequate vitamin A intake (10–14). Diarrhea and its interaction with malnutrition remain a leading cause of global DALYs (20). Severe diarrhea and undernutrition in the under-five population disrupt normal physiological maturation and delay achievement of key developmental benchmarks (19). Childhood wasting and stunting are risk factors for diarrhea, and diarrhea in turn may exacerbate wasting and stunting, creating a vicious cycle if not controlled promptly (18). Therefore, we selected childhood wasting and stunting as two key risk factors to examine their global distribution, temporal trends, decomposition, and future projections, to identify similarities and differences, and to support the development of better and more refined management strategies for childhood diarrhea, reducing the threat of diarrheal illness and death among children.

### Statistical analysis

Descriptive analysis: We tabulated crude and age-standardized measures (prevalence, DALYs, age-standardized prevalence, age-standardized DALY rates) according to year, sex, age, GBD region, and SDI region. All estimates are reported with 95% uncertainty intervals (UIs) alongside absolute counts and rates.

Temporal trend analysis: We computed EAPCs from age-standardized rates (ASRs). This widely adopted summary metric captures temporal variations in ASR trends over a defined period, enabling quantification of diarrhea burden trajectories attributable to individual risk factors. Age-standardized rates (per 100,000 population) were computed using the formula: $\mathrm{ASR}=\frac{\sum_{i\;=\;1}^{A}\;a_{i}w_{i}}{\sum_{i\;=\;1}^{A}\;w_{i}}\;\times 100000\;$

where $a_{i}$ denotes the case count in age category i, and $w_{i}$ refers to the population size (or weight) of the corresponding ith age subgroup in the selected standard reference population. The natural logarithm of the ASR was assumed to vary linearly over time: Y=α+βX+ε；where Y denotes ln(ASR),

X is the calendar year, and ε is the error term. Based on this model, β represents the positive or negative trend in the ASR. The EAPC was calculated as: EAPC=100times(exp(β)-1) with 95% confidence intervals (CIs) also derived from the linear regression model. A statistically significant increase in ASR was determined when both the EAPC value and its 95% CI lower bound were positive; in contrast, a significant decrease was identified if both the EAPC value and its 95% CI upper bound were negative.

- Decomposition analysis: We aimed to assess the contribution of key factors to alterations in DALYs linked to diarrhea induced by wasting and malnutrition between 1990 and 2021 through decomposition analysis. These alterations were split into two key components:
- Population growth: alterations caused entirely by an expansion in the overall population.
- Population aging: alterations stemming from modifications in population age distribution.
- Epidemiological changes: changes in disease burden resulting from epidemiological characteristics, effectiveness of prevention and control measures, advances in medical technology, or other non-demographic factors (excluding aging and population growth).

#### Burden projection

The ARIMA model was used to project diarrhea-related outcomes linked to wasting and stunting up to 2050. The optimal ARIMA model was screened according to three core parameters (*p*, *d*, *q*), in which *p* denotes the autoregressive order, *d* represents the degree of non-seasonal differencing, and *q* stands for the order of moving average terms from lagged prediction errors (23).

## Results

In 2021, deaths from wasting-related diarrhea among children aged <5 years were 129,466.84 (95% UI, 80,077.25–196,253.13), higher than those from stunting-related diarrhea: 89,545.67 (95% UI, 53,857.83–131,288.22). The mortality rate for wasting-related diarrhea was 19.67 (95% UI, 12.17–29.82) per 100,000 population, also higher than that for stunting-related diarrhea: 13.61 (95% UI, 8.18–19.95) per 100,000.

Boys had slightly higher mortality from wasting-related diarrhea (21.61 per 100,000; 95% UI, 12.73–35.37) than girls (17.6 per 100,000; 95% UI, 10.73–25.98) (Table 1). A similar pattern was observed for stunting-related diarrhea.

**Table 1:** Number of DALYs cases, the age-standardized DALYs rate/100,000, estimated annual percentage change (EAPC) of diarrhoeal diseases attributable to wasting in children under five in 1990 and 2021.

Region	Number of DALYs cases (95% UI) in 1990	The age-standardized DALYs rate/100,000 (95% UI) in 1990	Number of DALYs cases (95% UI) in 2021	The age-standardized DALYs rate/100,000 (95% UI) in 2021	EAPC (95% CI)
Global	64,052,886.81 (44,025,257.25–86,554,868.15)	10,332.15 (7,101.56–13,961.86)	11,592,673.48 (72,86,697.81–17,445,961.44)	1,761.34 (1,107.11–2,650.67)	−5.51 (−5.86 to −5.16)
Sex					
Both	64,052,886.81 (44,025,257.25–86,554,868.15)	10,332.15 (7,101.56–13,961.86)	11,592,673.48 (72,86,697.81–17,445,961.44)	1,761.34 (1,107.11–2,650.67)	−5.51 (−5.86 to −5.16)
Female	29,383,174.99 (19,534,821.12–41,472,361.91)	9,781.33 (6,502.92–13,805.68)	50,20,029.09 (31,14,790.75–73,55,923.87)	1,577.67 (978.9–2,311.78)	−5.66 (−6.07 to −5.24)
Male	34,669,711.82 (22,128,736.55–48,205,044.18)	10,849.97 (6,925.24–15,085.89)	65,72,644.39 (39,38,456.04–10,692,636.39)	1,933.25 (1,158.44–3,145.09)	−5.39 (−5.7 to −5.09)
Age					
<5 years	64,052,886.81 (44,025,257.25–86,554,868.15)	10,332.15 (7,101.56–13,961.86)	11,592,673.48 (72,86,697.81–17,445,961.44)	1,761.34 (1,107.11–2,650.67)	−5.51 (−5.86 to −5.16)
1–5 months	22,749,877.25 (15,656,772.68–29,755,023.97)	41,669.73 (28,677.67–54,500.68)	34,86,451.7 (21,85,393.49–52,22,601.9)	6,485.57 (4,065.32–9,715.2)	−5.9 (−6.21 to −5.59)
12–23 months	13,412,871.89 (86,32,064.46–19,274,981.86)	10,763.19 (6,926.82–15,467.26)	25,58,992.11 (15,76,867.52–39,51,873.86)	1,992.82 (1,227.99–3,077.53)	−5.36 (−5.69 to −5.02)
2–4 years	13,829,918.81 (91,35,498.54–19,519,742.31)	3,762.5 (2,485.36–5,310.45)	23,85,822.62 (14,57,925.38–37,99,167.1)	591.92 (361.71–942.57)	−5.75 (−6.13 to −5.38)
6–11 months	14,060,218.86 (90,59,538.5–19,821,877.4)	22,278.08 (14,354.62–31,407.3)	31,61,407.04 (18,33,348.53–49,59,519.5)	5,002.84 (2,901.22–7,848.3)	−4.65 (−4.96 to −4.34)
SDI region					
High-middle SDI	808,913.1 (492,725.22–11,83,719.85)	870.71 (530.37–1,274.15)	34,098.66 (23,103.19–48,817.12)	48.68 (32.98–69.69)	−9.29 (−9.43 to −9.16)
High SDI	46,874.27 (30,064.97–76,596.54)	75.96 (48.72–124.12)	5,607.39 (3,290.3–8,530)	10.41 (6.11–15.84)	−5.77 (−6.08 to −5.47)
Low-middle SDI	30,072,588.46 (21,068,749.12–40,281,223.75)	17,334.61 (12,144.57–23,219.13)	26,94,159.78 (16,37,904.52–43,74,310.39)	1,406.3 (854.95–2,283.31)	−7.63 (−7.99 to −7.27)
Low SDI	23,693,493.01 (14,841,756.31–32,624,455.95)	26,095.44 (16,346.35–35,931.78)	81,53,981.93 (49,53,284.97–12,358,606.59)	4,924.64 (2,991.56–7,464.04)	−5.18 (−5.51 to −4.85)
Middle SDI	93,96,630.97 (59,84,224.47–12,926,628.56)	4,685.76 (2,984.12–6,446.04)	695,938.7 (437,562.2–10,33,545.37)	394.04 (247.75–585.19)	−7.65 (−7.85 to −7.45)
GBD region					
Andean Latin America	188,454.5 (113,976.69–278,145.25)	3,568.2 (2,158.04–5,266.41)	8,578.48 (4,196.12–15,081.97)	139.36 (68.17–245)	−10.44 (−10.65 to −10.23)
Australasia	78 (53.83–108.89)	5.06 (3.49–7.06)	34.75 (24.07–52.33)	1.91 (1.33–2.88)	−0.41 (−1.46 to −0.65)
Caribbean	378,825.02 (241,802.38–539,882.78)	9,169.37 (5,852.77–13,067.73)	99,562.6 (52,512.54–170,462.34)	2,573.89 (1,357.55–4,406.78)	−3.81 (−4.28 to −3.33)
Central Asia	353,728.4 (215,375.33–501,789.3)	3,713.86 (2,261.27–5,268.38)	37,522.19 (19,488.3–63,887.96)	375.33 (194.94–639.07)	−8.36 (−8.73 to −8)
Central Europe	13,983.65 (8,102.64–20,252.54)	153.14 (88.73–221.79)	1,997.07 (1,089.21–3,065.06)	35.75 (19.5–54.87)	−4.78 (−5.79 to −3.76)
Central Latin America	988,797.3 (612,016.5–13,77,832.44)	4,295.77 (2,658.87–5,985.91)	59,057.59 (33,227.55–96,448.24)	293.96 (165.39–480.07)	−8.16 (−8.56 to −7.76)
Central Sub-Saharan Africa	23,90,840.68 (14,60,848.31–33,57,426.29)	23,023.38 (14,067.71–32,331.43)	400,583.71 (201,575.69–728,508.14)	1,901.52 (956.85–3,458.14)	−7.71 (−8.71 to −6.7)
East Asia	18,66,501.58 (10,39,086.03–29,18,446.82)	1,612.55 (897.71–2,521.36)	11,036.73 (6,918.41–16,768.3)	13.78 (8.64–20.94)	−15.63 (−16.14 to −15.11)
Eastern Europe	27,867.99 (20,136.43–36,461.47)	161.63 (116.79–211.47)	1,337.49 (971.87–1,812.03)	13.22 (9.6–17.91)	−9.18 (−9.84 to −8.52)
Eastern Sub-Saharan Africa	75,31,024.47 (42,00,087–11,432,481.74)	20,869.41 (11,638.96–31,680.83)	19,25,707.67 (11,17,610.71–31,36,274.33)	3,018.52 (1,751.84–4,916.07)	−6.18 (−6.37 to −5.98)
High-income Asia Pacific	4,023.06 (2,611.04–5,994.85)	39.38 (25.56–58.68)	1,367.15 (294.46–2,813.28)	21.19 (4.56–43.6)	−1.18 (−1.55 to −0.81)
High-income North America	2,854.07 (1,906.09–4,077.98)	13.16 (8.79–18.81)	800.84 (466.96–1,192.16)	3.91 (2.28–5.82)	−3.98 (−4.55 to −3.4)
North Africa and Middle East	27,88,577.76 (17,66,474.74–42,38,149.18)	5,443.29 (3,448.15–8,272.85)	353,491.71 (211,684.83–634,184.33)	578.2 (346.25–1,037.32)	−7.26 (−7.64 to −6.87)
Oceania	73,206.9 (42,548.97–119,465.14)	7,290.18 (4,237.16–11,896.72)	63,161.76 (31,287.07–108,369.84)	3,265.1 (1,617.36–5,602.1)	−1.95 (−2.23 to −1.68)
South Asia	28,262,501.7 (20,623,088.9–37,516,277.65)	17,998.64 (13,133.57–23,891.8)	22,58,131.99 (12,06,547.04–37,21,504.37)	1,423.85 (760.78–2,346.56)	−7.6 (−8 to −7.21)
Southeast Asia	64,84,868.05 (38,25,987.65–90,95,898.16)	11,125.25 (6,563.75–15,604.66)	414,211.17 (270,178.22–623,332.46)	735.92 (480.02–1,107.47)	−8.56 (−8.64 to −8.48)
Southern Latin America	13,321.32 (8,155.78–19,039.63)	258.82 (158.46–369.92)	831.06 (534.07–1,237.51)	19.42 (12.48–28.92)	−7.37 (−7.7 to −7.04)
Southern Sub-Saharan Africa	624,857.38 (381,693.19–903,567.74)	8,361.64 (5,107.7–12,091.26)	178,491.61 (95,704.32–295,727.31)	2,223.06 (1,191.97–3,683.19)	−4.04 (−4.69 to −3.38)
Tropical Latin America	730,287.12 (431,823.56–10,37,768.95)	4,275.89 (2,528.36–6,076.22)	12,094 (7,281.78–18,295.96)	70.28 (42.32–106.32)	−12.77 (−12.99 to −12.55)
Western Europe	3,168.93 (1,226.41–5,829.84)	13.8 (5.34–25.39)	1,844.36 (782.27–3,389.49)	8.69 (3.68–15.97)	−0.73 (−1.28 to −0.16)
Western Sub-Saharan Africa	11,325,118.91 (70,24,209.11–15,857,597.3)	31,683.64 (19,651.23–44,363.9)	57,62,829.54 (34,10,559.71–90,22,153.73)	7,207.31 (4,265.43–11,283.6)	−4.53 (−4.94 to −4.11)

In 2021, age-standardized DALYs from wasting- and stunting-related diarrhea among children aged <5 years were 11,592,673.48 (95% UI, 7,286,697.81–17,445,961.44; Table 1) and 7,979,457.04 (95% UI, 4,714,036.18–11,643,869.86; Table 2), respectively. Age-standardized DALY rates were 1,761.34 (95% UI, 1,107.11–2,650.67) and 1,212.37 (95% UI, 716.23–1,769.12) per 100,000 population, respectively.

**Table 2:** Number of DALYs cases, the age-standardized DALYs rate/1,00,000, estimated annual percentage change (EAPC) of diarrhoeal diseases attributable to stunting in children under five in 1990 and 2021.

Region	Number of deaths cases (95% UI) in 1990	The age-standardized deaths rate/1,00,000 (95% UI) in 1990	Number of deaths cases (95% UI) in 2021	The age-standardized deaths rate/1,00,000 (95% UI) in 2021	EAPC (95% CI)
Global	7,16,508.62 (4,87,965.26–9,72,261.07)	115.58 (78.71–156.83)	1,29,466.84 (80,077.25–1,96,253.13)	19.67 (12.17–29.82)	−5.52 (−5.87 to −5.17)
Sex					
Both	7,16,508.62 (4,87,965.26–9,72,261.07)	115.58 (78.71–156.83)	1,29,466.84 (80,077.25–1,96,253.13)	19.67 (12.17–29.82)	−5.52 (−5.87 to −5.17)
Female	3,28,590.22 (2,14,137.61–4,66,395.14)	109.38 (71.28–155.26)	55,992.94 (34,136.94–82,669.59)	17.6 (10.73–25.98)	−5.67 (−6.08 to −5.26)
Male	3,87,918.4 (2,45,061.32–5,41,161.98)	121.4 (76.69–169.36)	73,473.91 (43,283.34–1,20,258.15)	21.61 (12.73–35.37)	−5.4 (−5.7 to −5.1)
Age					
<5 years	7,16,508.62 (4,87,965.26–9,72,261.07)	115.58 (78.71–156.83)	1,29,466.84 (80,077.25–1,96,253.13)	19.67 (12.17–29.82)	−5.52 (−5.87 to −5.17)
1–5 months	2,52,891.69 (1,73,590.98–3,30,744.74)	463.21 (317.96–605.81)	38,662.05 (24,027.81–58,148.67)	71.92 (44.7–108.17)	−5.91 (−6.22 to −5.6)
12–23 months	1,50,293 (95,794.75–2,17,404.58)	120.6 (76.87–174.46)	28,658.62 (17,573.01–44,402.23)	22.32 (13.68–34.58)	−5.36 (−5.69 to −5.03)
2–4 years	1,56,536.53 (1,00,519.27–2,23,866.3)	42.59 (27.35–60.9)	26,923.21 (15,679.79–43,621.3)	6.68 (3.89–10.82)	−5.78 (−6.15 to −5.41)
6–11 months	1,56,787.39 (1,00,272.84–2,21,773.13)	248.43 (158.88–351.39)	35,222.96 (20,191.81–55,458.23)	55.74 (31.95–87.76)	−4.66 (−4.96 to −4.34)
SDI region					
High-middle SDI	8,852.07 (5,019.19–13,253.83)	9.53 (5.4–14.27)	351.49 (205.46–533.65)	0.5 (0.29–0.76)	−9.49 (−9.64 to −9.35)
High SDI	466.99 (235.09–833.29)	0.76 (0.38–1.35)	39.83 (20.53–60.81)	0.07 (0.04–0.11)	−6.93 (−7.29 to −6.57)
Low-middle SDI	3,36,450.69 (2,32,600.97–4,52,672.82)	193.94 (134.08–260.93)	29,860.88 (17,823.32–48,918.63)	15.59 (9.3–25.53)	−7.66 (−8.02 to −7.31)
Low SDI	2,66,083.71 (1,65,693.25–3,67,793.11)	293.06 (182.49–405.08)	91,539.96 (55,261.47–1,39,445.19)	55.29 (33.38–84.22)	−5.18 (−5.51 to −4.85)
Middle SDI	1,04,271.04 (65,007.74–1,44,787.34)	52 (32.42–72.2)	7,575.73 (4,470.81–11,515.24)	4.29 (2.53–6.52)	−7.71 (−7.9 to −7.51)
GBD region					
Andean Latin America	2,069.7 (1,191.8–3,102.93)	39.19 (22.57–58.75)	93.5 (41.92–168.64)	1.52 (0.68–2.74)	−10.47 (−10.7 to −10.24)
Australasia	0.64 (0.3–1.04)	0.04 (0.02–0.07)	0.3 (0.13–0.5)	0.02 (0.01–0.03)	0.11 (−1.29 to −1.52)
Caribbean	4,243.06 (2,691.06–6,061.36)	102.7 (65.14–146.71)	1,114.67 (584.23–1,913.8)	28.82 (15.1–49.48)	−3.81 (−4.29 to −3.33)
Central Asia	3,928.62 (2,352.78–5,609.77)	41.25 (24.7–58.9)	417.74 (214.18–716.28)	4.18 (2.14–7.16)	−8.37 (−8.73 to −8)
Central Europe	152.31 (83.15–224.94)	1.67 (0.91–2.46)	21.7 (11.03–33.94)	0.39 (0.2–0.61)	−4.81 (−5.88 to −3.72)
Central Latin America	11,014.39 (6,688.43–15,443.42)	47.85 (29.06–67.09)	651.38 (349.25–1,077.13)	3.24 (1.74–5.36)	−8.18 (−8.59 to −7.77)
Central Sub-Saharan Africa	26,772.24 (16,221.19–37,699.83)	257.81 (156.21–363.04)	4,459.48 (2,185.57–8,182.03)	21.17 (10.37–38.84)	−7.73 (−8.73 to −6.72)
East Asia	20,616.74 (11,035–32,702.55)	17.81 (9.53–28.25)	107.12 (53.7–180.38)	0.13 (0.07–0.23)	−15.98 (−16.51 to −15.46)
Eastern Europe	273.17 (158.18–386.48)	1.58 (0.92–2.24)	12.05 (6.53–18.07)	0.12 (0.06–0.18)	−9.75 (−10.46 to −9.02)
Eastern Sub-Saharan Africa	84,433.56 (46,650.1–1,28,779.22)	233.98 (129.27–356.86)	21,594.13 (12,459.62–35,351.11)	33.85 (19.53–55.41)	−6.18 (−6.37 to −5.99)
High-income Asia Pacific	28.58 (14.09–46.37)	0.28 (0.14–0.45)	5.41 (2.79–8.38)	0.08 (0.04–0.13)	−2.86 (−3.26 to −2.46)
High-income North America	22.72 (11.07–34.21)	0.1 (0.05–0.16)	8.34 (4.03–13.04)	0.04 (0.02–0.06)	−3.35 (−3.97 to −2.74)
North Africa and Middle East	30,930.73 (19,241.11–47,432.04)	60.38 (37.56–92.59)	3,889.88 (2,258.05–7,082.68)	6.36 (3.69–11.58)	−7.31 (−7.7 to −6.91)
Oceania	815.29 (467.81–1,337.97)	81.19 (46.59–133.24)	701.84 (338.83–1,214.47)	36.28 (17.52–62.78)	−1.95 (−2.23 to −1.68)
South Asia	3,16,309.98 (2,29,273.13–4,21,954.82)	201.44 (146.01–268.72)	24,972.14 (12,930.71–41,605)	15.75 (8.15–26.23)	−7.64 (−8.04 to −7.25)
Southeast Asia	72,241.32 (42,090.23–1,02,062.65)	123.94 (72.21–175.1)	4,454.53 (2,715.41–6,923.85)	7.91 (4.82–12.3)	−8.67 (−8.75 to −8.58)
Southern Latin America	139.34 (72.54–208.57)	2.71 (1.41–4.05)	8.21 (4.03–13.47)	0.19 (0.09–0.31)	−7.67 (−8 to −7.35)
Southern Sub-Saharan Africa	6,946.96 (4,174.73–10,115.01)	92.96 (55.86–135.36)	1,991.78 (1,055.65–3,316.04)	24.81 (13.15–41.3)	−4.02 (−4.68 to −3.37)
Tropical Latin America	8,095.99 (4,701.65–11,577.29)	47.4 (27.53–67.79)	128.26 (69.15–203.01)	0.75 (0.4–1.18)	−12.86 (−13.1 to −12.63)
Western Europe	17.28 (8.55–26.08)	0.08 (0.04–0.11)	10.12 (5.09–15.68)	0.05 (0.02–0.07)	−0.27 (−0.98 to −0.46)
Western Sub-Saharan Africa	1,27,455.99 (78,454.25–1,78,856.86)	356.58 (219.49–500.38)	64,824.28 (38,172.27–1,01,641.13)	81.07 (47.74–127.12)	−4.53 (−4.95 to −4.12)

The age-standardized DALY rate from wasting-related diarrhea was higher in males (1,933.25; 95% UI, 1,158.44–3,145.09) than in females (1,577.67; 95% UI, 978.9–2,311.78). Between 1990 and 2021, the age-standardized DALY rate per 100,000 decreased slightly more in females (EAPC = −5.66; 95% CI, −6.07 to −5.24) relative to males (EAPC = −5.39; 95% CI, −5.70 to −5.09). A comparable trend was identified for diarrhea associated with stunting.

**Figure 1:**
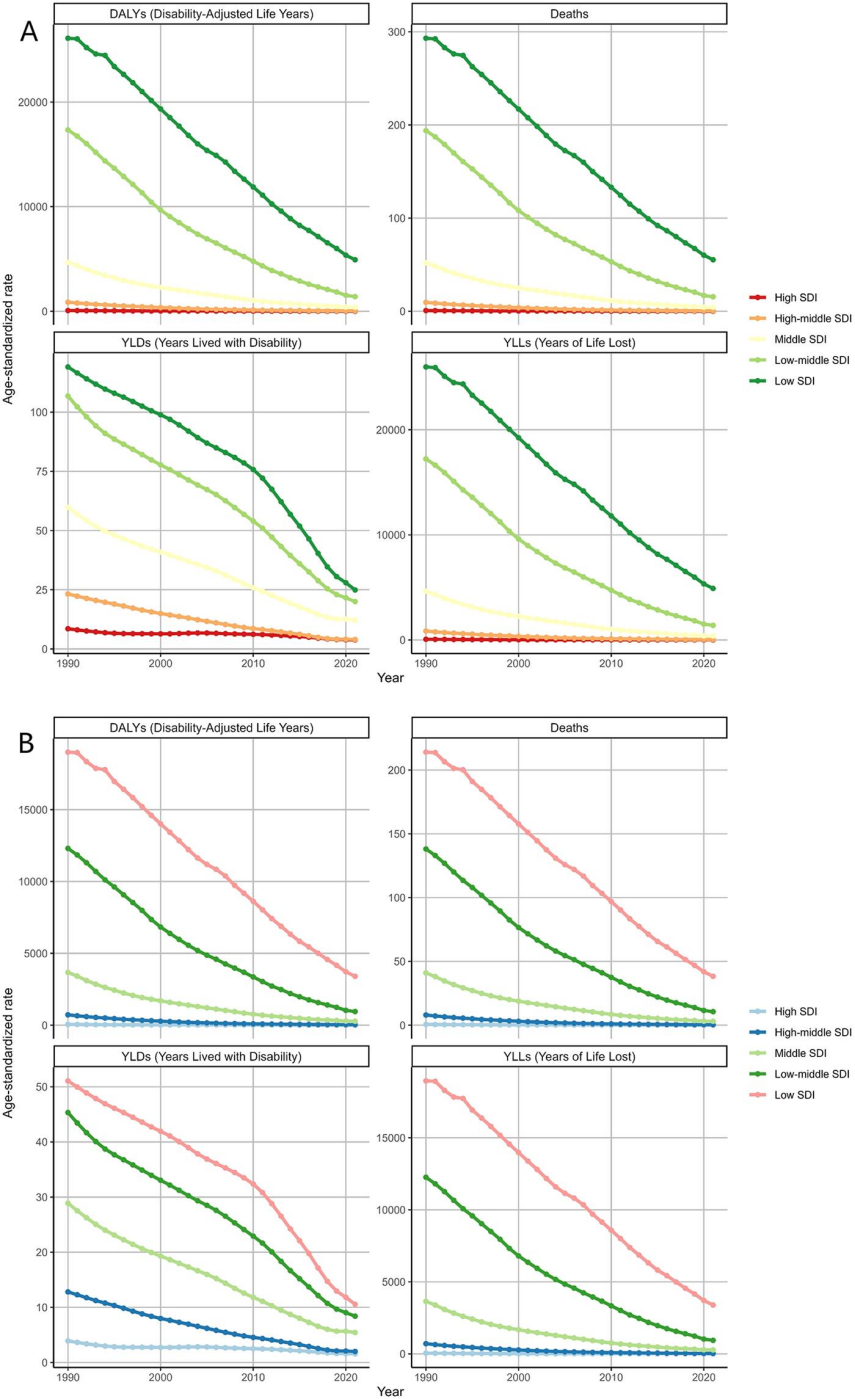
Trends in age-standardized DALYs, deaths, YLDs, and YLLs of diarrhea per 100,000 children under 5 attributable to nutritional risk factors, by SDI region, 1990–2021. **(A)** Wasting; **(B)** stunting.

**Figure 2:**
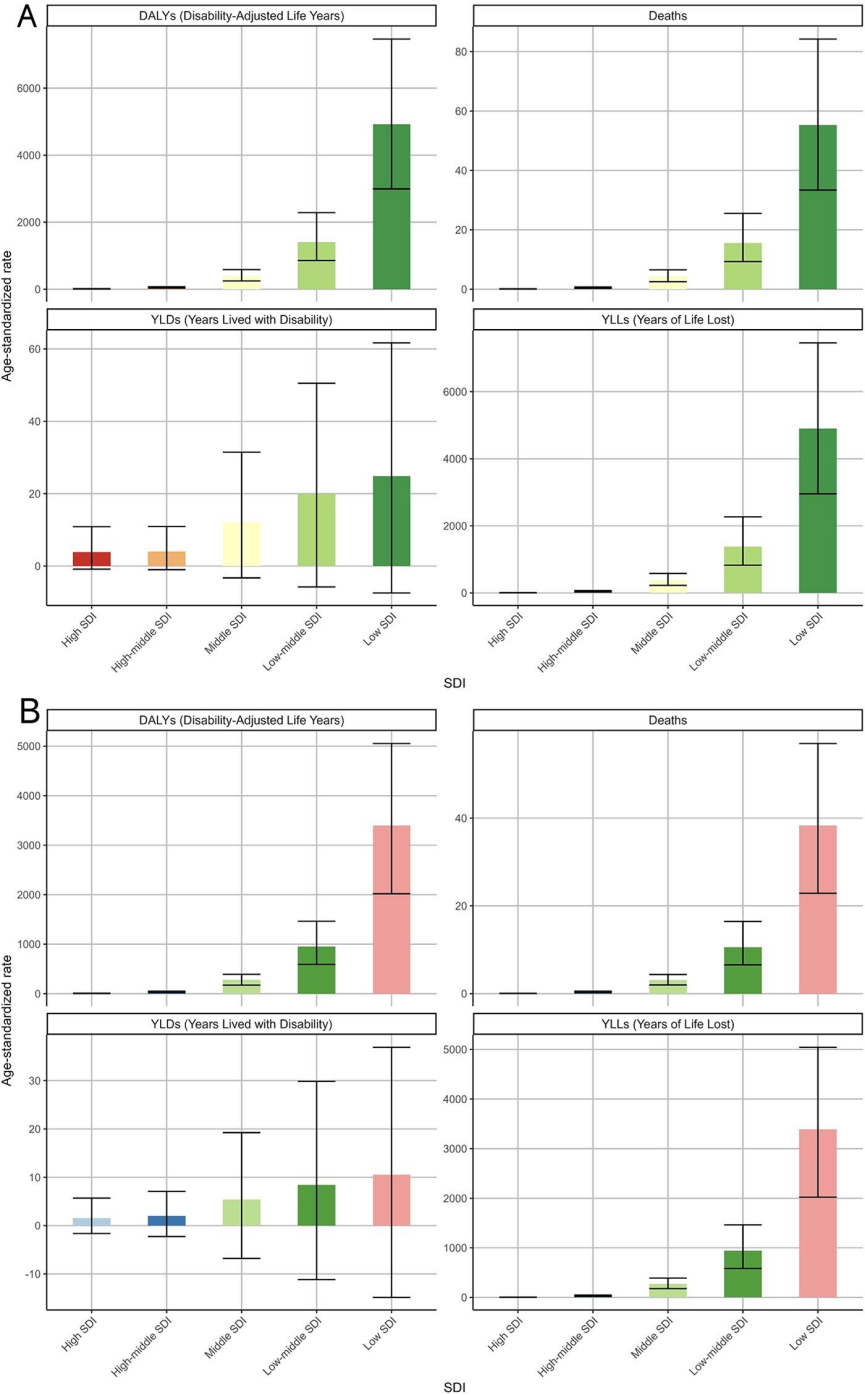
Age-standardized DALYs, deaths, YLDs, and YLLs of diarrhea per 100,000 under-5 children attributable to nutritional risk factors by SDI regions, 2021. **(A)** Wasting; **(B)** stunting.

Between 1990 and 2021, DALYs, deaths, YLDs, and YLLs attributable to wasting- and stunting-related diarrhea showed overall steady downward trends (Figure 1). In 2021, among the five SDI quintiles, the low SDI group had the highest age-standardized DALY rates from wasting and stunting (4,924.64; 95% UI 2,991.56–7,464.04 and 3,400.89; 95% UI 2,019.22–5,053.71, respectively), followed by the low-middle SDI group (1,406.3; 95% UI 854.95–2,283.31 and 950.11; 95% UI 592.35–1,463.41). The high SDI group had the lowest burden (10.41; 95% UI, 6.11–15.84 and 6.64; 95% UI, 2.15–10.03) (Figure 2).

From 1990 to 2021, the overall DALYs, deaths, YLDs, and YLLs due to diarrhea caused by wasting and stunting showed a steady downward trend. In 2021, among the five SDI quintiles, the low SDI region had the highest age-standardized DALY rates of diarrhea caused by wasting and stunting (4,924.64 [2,991.56–7,464.04] and 3,400.89 [2,019.22–5,053.71]), followed by the low-middle SDI region (1,406.3 [854.95–2,283.31] and 950.11 [592.35–1,463.41]), while the high SDI region had the lowest disease burden (10.41 [6.11–15.84] and 6.64 [2.15–10.03]). From 1990 to 2021, the middle-high SDI region had the largest decrease in the age-standardized DALY rate of diarrhea caused by wasting and stunting (EAPC −9.29 [−9.43 to 9.16] and −9.59 [−9.74 to 9.45], respectively). The two regions with the smallest decrease in the DALY rate of diarrhea caused by wasting were the low SDI region (EAPC −5.18 [−5.51 to 4.85]) and the high SDI region (EAPC −5.77 [−6.08 to 5.47]). For diarrhea caused by stunting, the region with the smallest decrease was the low SDI region (EAPC −5.31 [−5.67 to 4.94]) (Tables 1, 2).

### GBD regional description

**Figure 3:**
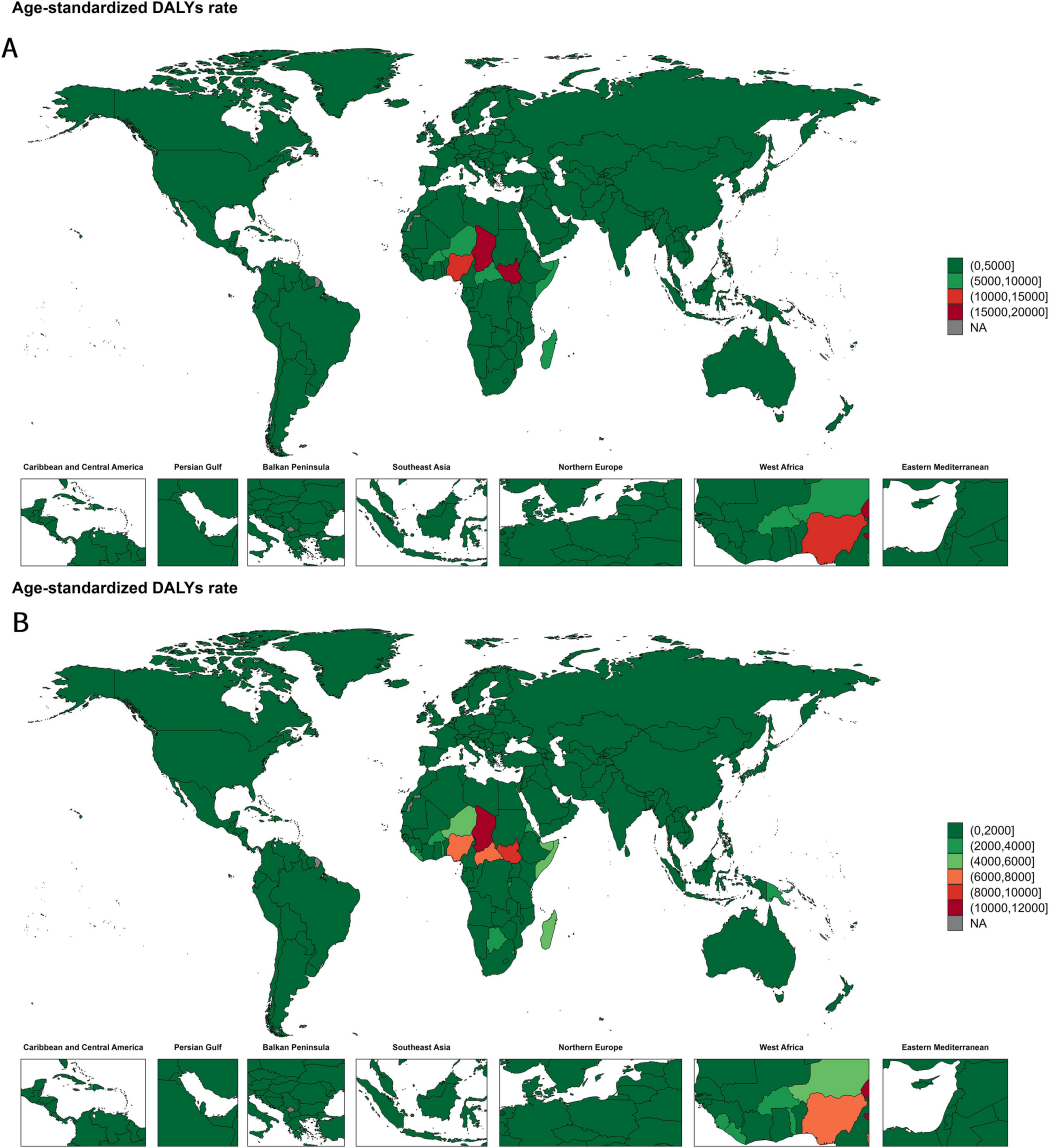
World map of age-standardized DALYs rate for diarrheal diseases by risk factor, 2021. **(A)** Wasting; **(B)** stunting.

**Figure 4:**
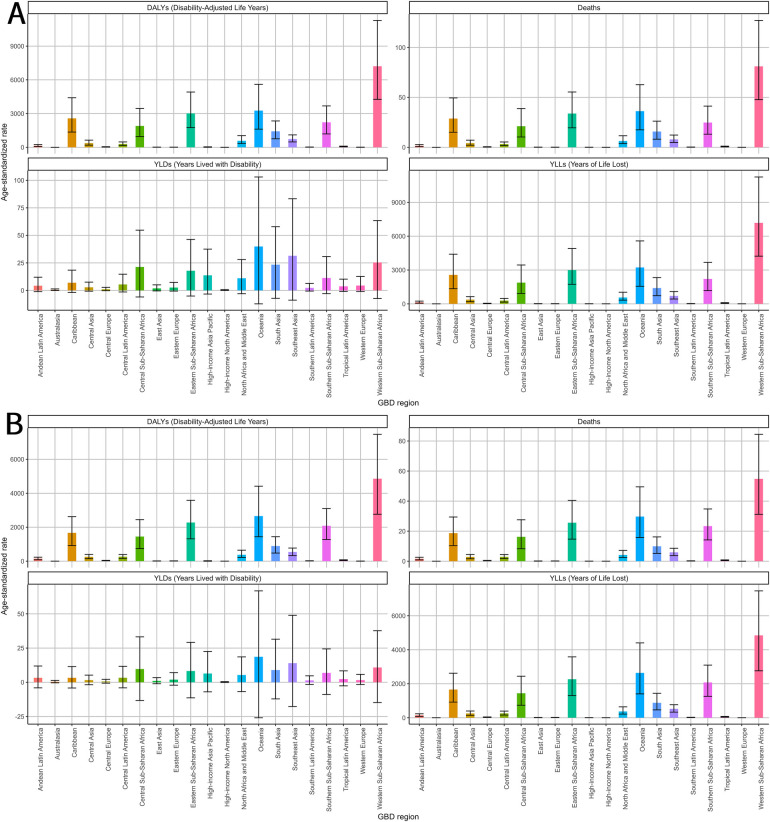
Regional distribution of age-standardized rates of DALYs, deaths, YLDs, and YLLs from diarrheal diseases by risk factor in 2021, GBD regions. **(A)** Wasting; **(B)** stunting.

In 2021, the highest age-standardized DALY rates from wasting- and stunting-related diarrhea were in Western Sub-Saharan Africa (7,207.31; 95% UI, 4,265.43–11,283.6 and 4,855.21; 95% UI, 2,764.34–7,469.98, respectively) (Tables 1, 2), especially in Uganda and Chad (Figure 3). In 2021, the global number of DALY cases from wasting-related diarrhea was 11,592,673.48 (95% UI, 7,286,697.81–17,445,961.44), and the number in Western Sub-Saharan Africa was 5,762,829.54 (95% UI, 3,410,559.71–9,022,153.73), accounting for approximately half of the global total. The number of DALY cases from stunting-related diarrhea was 7,979,457.04 (95% UI, 4,714,036.18–11,643,869.86), mainly concentrated in Western Sub-Saharan Africa. In 2021, regions with the highest age-standardized DALYs, deaths, and YLLs were all in Western Sub-Saharan Africa, while the highest YLD rates were in Oceania, followed by Southeast Asia (Figure 4). This may reflect relatively weak health systems, limited access to safe drinking water and sanitation, and poor healthcare access due to geographic isolation in Oceania. In Western Sub-Saharan Africa, high mortality may mean many patients die before developing long-term disability, which does not imply better performance than regions such as Oceania.

From 1990 to 2021, the fastest declines in age-standardized mortality from wasting- and stunting-related diarrhea in children under 5 years old were in East Asia (EAPC = −15.98; 95% CI, −16.51 to −15.46 and −16.24; 95% CI, −16.77 to −15.70, respectively) and Tropical Latin America (EAPC = −12.86; 95% CI, −13.10 to −12.63 and −12.92; 95% CI, −13.17 to −12.67, respectively). The most substantial absolute decreases in diarrhea mortality from wasting and stunting were in Western Sub-Saharan Africa. In Western Sub-Saharan Africa, mortality from wasting-related diarrhea decreased by 275 per 100,000 (from 356 to 81).

The fastest declines in age-standardized DALYs from wasting- and stunting-related diarrhea were in East Asia (EAPC = −15.63; 95% CI, −16.14 to −15.11 and −16.02; 95% CI, −16.55 to −15.48, respectively) and Tropical Latin America (EAPC = −12.77; 95% CI, −12.99 to −12.55 and −12.85; 95% CI, −13.09 to −12.61, respectively). The magnitudes of reduction in age-standardized mortality and DALYs were similar between 1990 and 2021.

### Age stratification

Children in different age groups have varying susceptibility and immunity due to developmental differences. We divided children under 5 years old into four groups: 1–5 months, 6–11 months, 12–23 months, and 2–4 years to examine the age-specific effects of wasting on diarrheal burden.

**Figure 5:**
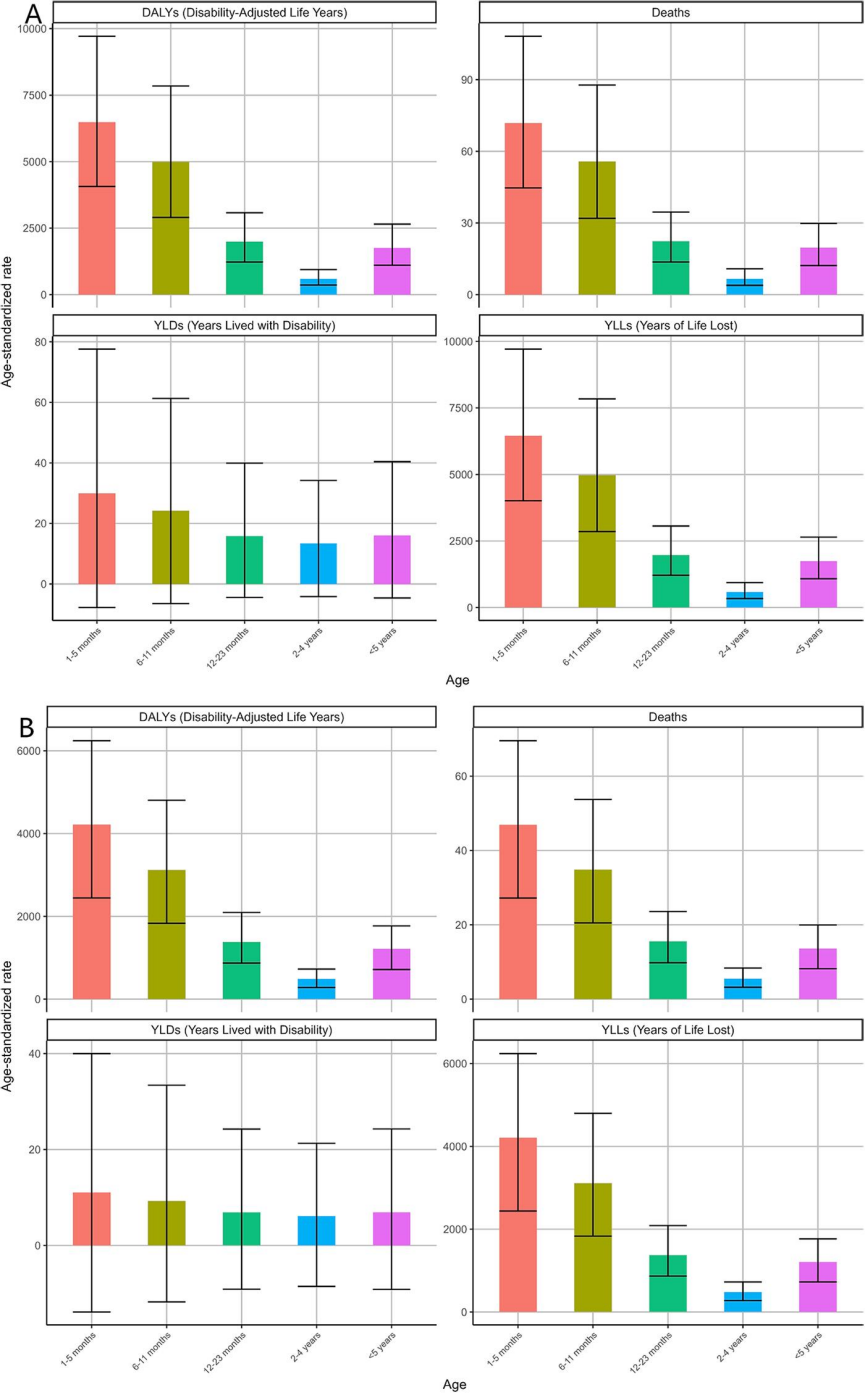
Age distribution of age-standardized rates of DALYs, deaths, YLDs, and YLLs from diarrheal diseases attributable to different risk factors in 2021. **(A)** Wasting; **(B)** stunting.

In 2021, age-standardized DALY rates from wasting- and stunting-related diarrhea were highest at age 1–5 months (6,485.57; 95% UI, 4,065.32–9,715.2 and 4,219.4; 95% UI, 2,444.16–6,244.13, respectively) and lowest at age 2–4 years (591.92; 95% UI, 361.71–942.57 and 486.42; 95% UI, 281.97–725.51, respectively). Age-standardized DALYs, deaths, YLDs, and YLLs all decreased with age, indicating that the impacts of wasting and stunting on diarrheal burden diminish with increasing age (Figure 5).

**Figure 6:**
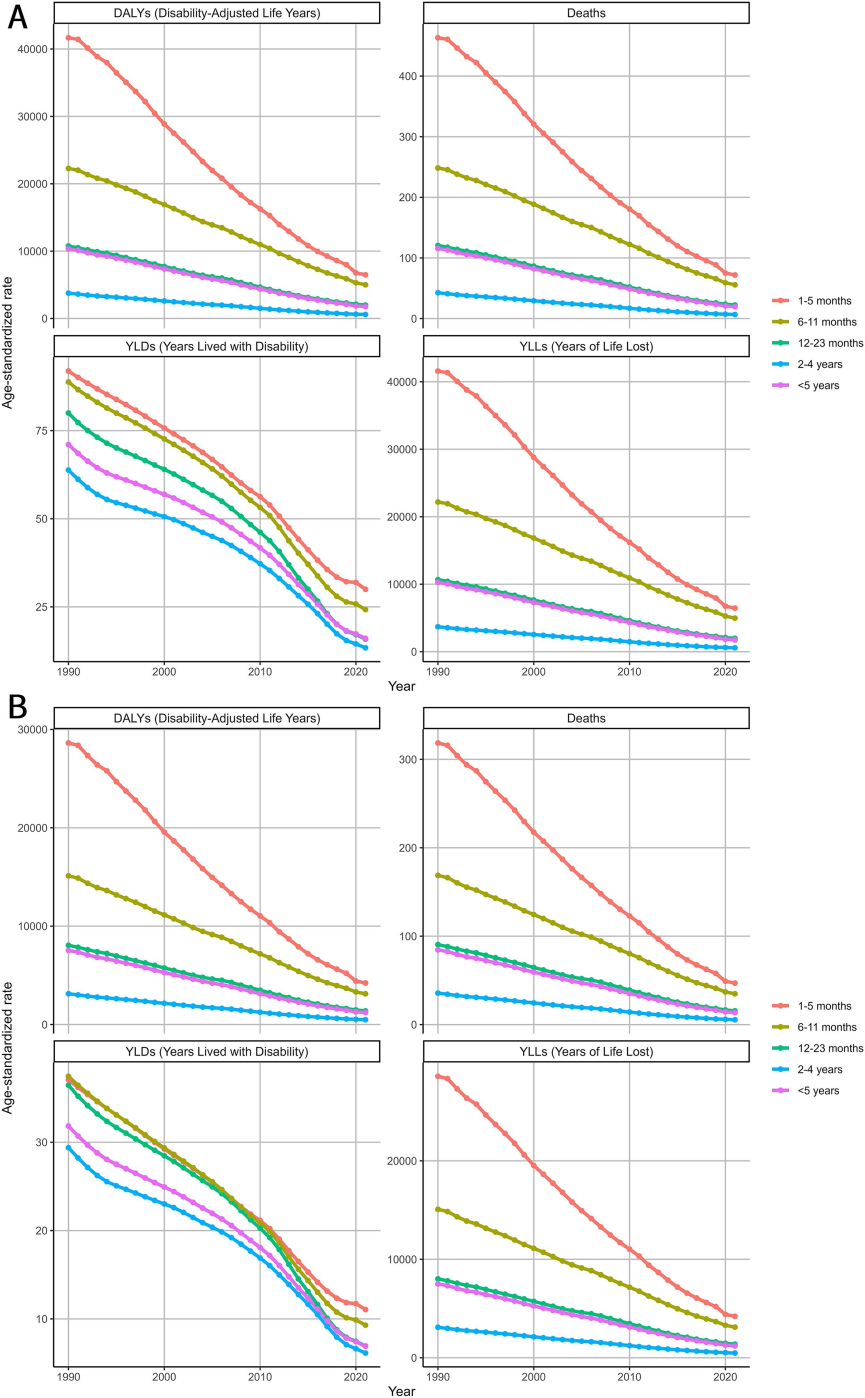
Trends in age-standardized rates of DALYs, deaths, YLDs, and YLLs from diarrheal diseases attributable to different risk factors, by age group, 1990–2021. **(A)** Wasting; **(B)** stunting.

Between 1990 and 2021, DALYs, deaths, YLDs, and YLLs from wasting- and stunting-related diarrhea decreased gradually with age in all groups (Figure 6). The smallest reduction in age-standardized DALYs was in the 6–11 month group, with EAPC = −4.65 (95% CI, −4.96 to −4.34) for wasting and −4.88 (95% CI, −5.21 to −4.55) for stunting.

### Country-specific description

**Figure 7:**
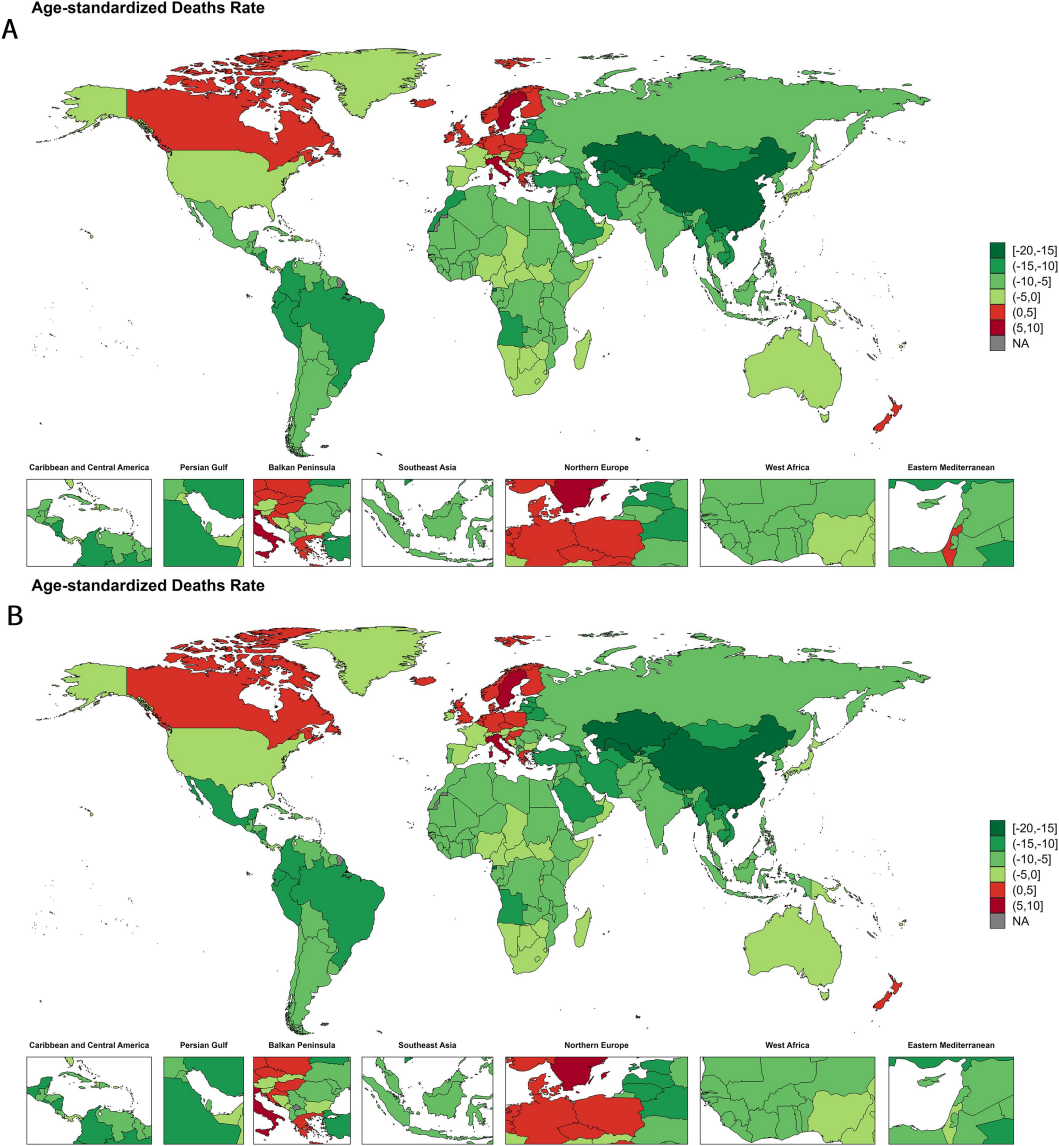
Global maps of EAPC in age-standardized diarrheal disease death rate by risk factor，1990–2021. **(A)** Wasting; **(B)** stunting.

From 1990 to 2021, global deaths from wasting-related diarrhea in children under 5 years old decreased significantly from 716,508.62 (95% UI, 487,965.26–972,261.07) to 129,466.84 (95% UI, 80,077.25–196,253.13). Age-standardized mortality also declined from 115.58 (95% UI, 78.71–156.83) to 19.67 (95% UI, 12.17–29.82) per 100,000 population (EAPC = −5.52; 95% CI, −5.87 to −5.17). The largest declines were in Uzbekistan (EAPC = −16.22; 95% CI, −16.95 to −15.48) and China (EAPC = −16.20; 95% CI, −16.74 to −15.65). However, age-standardized mortality increased in some countries, including Sweden (EAPC = 8.19; 95% CI, 6.85–9.54), Italy (EAPC = 6.25; 95% CI, 4.33–8.20), Canada (EAPC = 4.67; 95% CI, 3.04–6.34), and Greece (EAPC = 3.38; 95% CI, 2.69–4.07) (Figure 7). Age-standardized mortality from stunting-related diarrhea also declined, with similar countries showing the largest reductions and similar countries showing increases.

### SDI-stratified description

In 2021, the high SDI region had relatively low deaths and DALYs from wasting-related diarrhea: 39.83 (95% UI 20.53–60.81) and 5,607.39 (95% UI, 3,290.3–8,530), respectively. Age-standardized mortality decreased (EAPC = −6.93; 95% CI, −7.29 to −6.57), and age-standardized DALYs decreased (EAPC = −5.77; 95% CI, −6.08 to −5.47). The low SDI region had high deaths, decreasing from 266,083.71 (95% UI, 165,693.25–367,793.11) in 1990 to 91,539.96 (95% UI, 55,261.47–139,445.19) in 2021 (Table 1). Age-standardized mortality had an EAPC of −5.18 (95% CI, −5.51 to −4.85), and age-standardized DALYs had an EAPC of −5.18 (95% CI, −5.51 to −4.85).

The high-middle SDI region showed the largest reductions in age-standardized mortality (EAPC = −9.49; 95% CI, −9.64 to −9.35) and age-standardized DALYs (EAPC = −9.29; 95% CI, −9.43 to −9.16). The low SDI region had the smallest reductions in age-standardized DALYs and mortality, followed by the high SDI region.

In 2021, the lowest deaths and DALYs from stunting-related diarrhea were also in the high SDI region: 30.58 (95% UI, 18.72–42.72) and 3,576.31 (95% UI, 1,158.26–5,398.09), respectively. Age-standardized mortality decreased (EAPC = −6.97; 95% CI, −7.32 to −6.62), and age-standardized DALYs decreased (EAPC = −6.29; 95% CI, −6.61 to −5.98). The high-middle SDI region also showed the largest reductions, and the low SDI region the smallest.

### DALY rate decomposition

**Figure 8:**
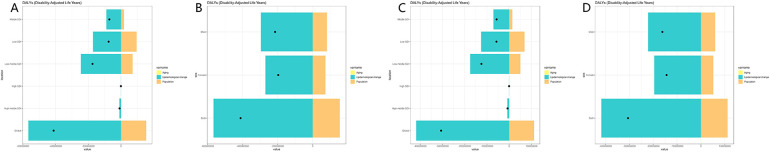
Decomposition analysis of age-standardized DALY rates for diarrheal disease attributable to different risk factors. **(A,B)** Wasting; **(C,D)** stunting.

From 1990 to 2021, global DALYs decreased. Worldwide, epidemiological changes were the main factor reducing the burden of wasting-related diarrhea, accounting for 137.53% of the DALY reduction. This pattern was consistent across SDI regions. The low SDI region had the largest percentage contribution from epidemiological changes (−226.08%), and the low-middle SDI quintile had the largest absolute reduction. Given that all participants were under-5 children, population aging had little impact. Globally, population growth increased DALYs by 37.53%. Regions with low SDI exhibited the highest relative rise attributable to population growth (126.08%). High and high-middle SDI regions showed similar percentage changes to the global average, although the absolute magnitudes were smaller. The effects of epidemiological changes and population growth on DALYs were slightly greater in males compared with females (Figure 8), but percentages were similar. Decomposition analysis for stunting-related diarrhea showed a generally similar pattern to wasting-related diarrhea.

### Health inequality analysis

**Figure 9:**
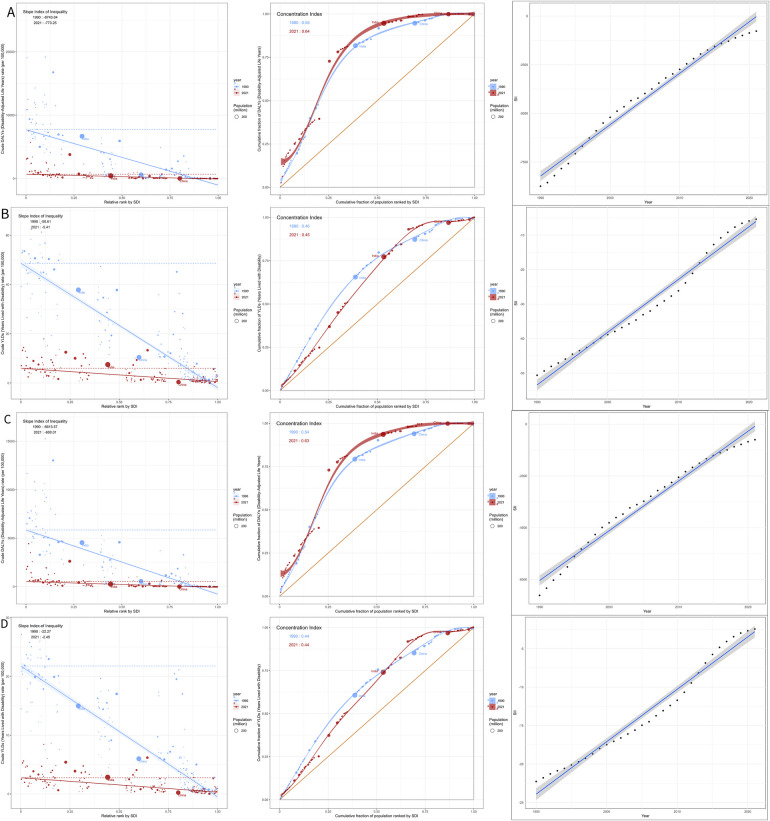
Health inequality in diarrheal disease by risk factors: wasting **(A,B)** and stunting **(C,D)**.

The absolute health inequality index for wasting-related diarrhea decreased substantially from −8,743.04 in 1990 to −773.25 in 2021 (a reduction of approximately 91%). The concentration index increased from 0.55 in 1990 to 0.64 in 2021, and the SII increased (Figure 9A). A crossover between 1990 and 2021 occurred at approximately SDI = 0.5, suggesting that the benefits of global health improvement have not been equally shared: wealthier populations accelerated health gains beyond the crossover point, while poorer populations remained at low survival levels to the left of the crossover (Figure 9B). Health inequality analyses for stunting-related diarrhea showed generally similar trends (Figure 9).

### Projection and analysis

Forecasts of the diarrheal disease burden attributable to wasting from 2022 to 2050 show that the ARIMA model predicts annual declines in both deaths and disability-adjusted life years (DALYs) due to the disease burden, with a greater magnitude of decline among females than males.

**Figure 10:**
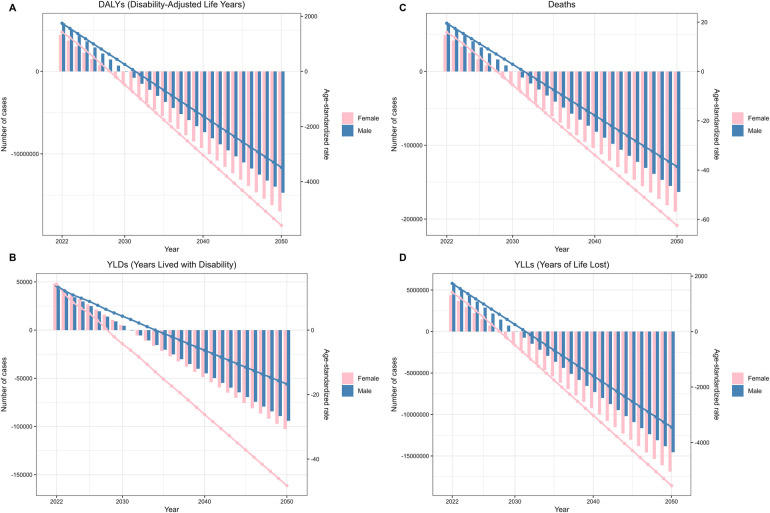
Predicted burden of diarrheal disease attributable to wasting from 2022 to 2050: Results of the ARIMA model. Panels **A**, **B**, **C**, and **D** represent DALYs, YLDs, deaths, and YLLs, respectively.

For males, the predicted number of DALYs due to wasting-related diarrheal disease from 2022 to 2050 will decrease from 5,838,984.85628718 (6,155,963.7017304, 5,522,006.01084395) to −14,703,482.0867518 (−4,173,388.2774463, −25,233,575.8960572), representing a reduction of approximately 20,542,467. For females, the predicted number of DALYs due to wasting-related diarrheal disease will decrease from 4,434,973.23849447 (4,741,908.48151057, 4,128,037.99547838) to −16,975,662.5953958 (−14,005,122.1798611, −19,946,203.0109305), representing a reduction of approximately 21,410,636 (Figure 10).

**Figure 11:**
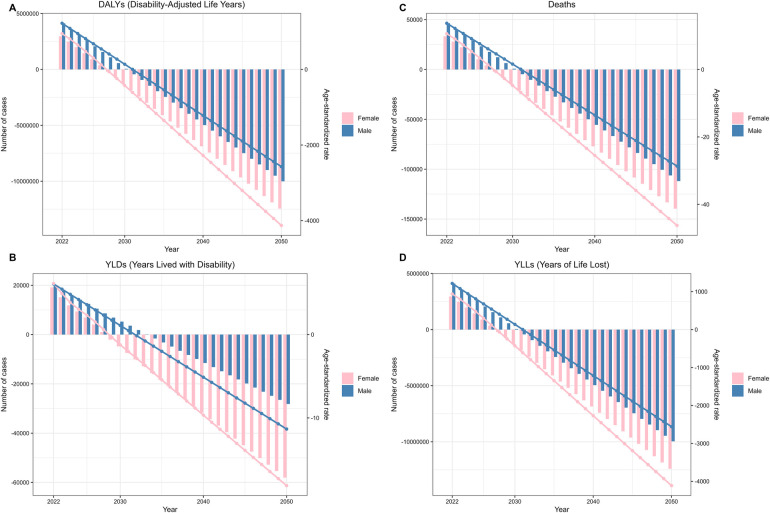
Predicted burden of diarrheal disease attributable to stunting from 2022 to 2050: ARIMA model. Panels **A**, **B**, **C**, and **D** represent DALYs, YLDs, deaths, and YLLs, respectively.

Forecasts of the diarrheal disease burden attributable to stunting from 2022 to 2050 show that the ARIMA model also predicts annual declines in both deaths and DALYs, with a greater decline among females than males. For females, the burden decreased from 2,967,240.77055707 (3,190,300.02337962, 2,744,181.51773452) in 2022 to −12,440,259.4768744 (−9,941,474.64205446, −14,939,044.3116943) in 2050, a reduction of 15,407,499. For males, the burden decreased from 4,129,210.71693671 (4,335,286.00418428, 3,923,135.42968914) in 2022 to −10,004,893.5293255 (−1,529,456.40136079, −18,480,330.6572901), a reduction of 14,134,104 (Figure 11). The reduction in diarrheal disease attributable to wasting is greater than that attributable to stunting.

## Discussion

Based on the GBD 2021 database, this study innovatively explored the long-term changing patterns of under-five diarrheal disease burden linked to wasting and stunting across the globe during 1990–2021. It filled the research gap regarding the combined effects of the two key nutritional determinants. With the application of decomposition analysis, we quantified the independent contributions of epidemiological transitions and demographic expansion to nutrition-relevant diarrheal burden. Notably, we identified a critical dilemma in low SDI settings, where the benefits of epidemiological optimization were largely counterbalanced by population growth. Additionally, this study delivered long-term burden projections up to 2050, offering reliable evidence for evidence-based childhood diarrhea prevention and control in the coming decades.

A bidirectional association exists between malnutrition and diarrhea: wasting and stunting act as independent risk factors for childhood diarrhea, and conversely, diarrheal episodes further worsen nutritional deficits. Therefore, evaluating the disease burden of diarrhea attributable to wasting and stunting is essential for developing tailored prevention strategies. In the present study, we first summarized the spatiotemporal distribution features of diarrheal burden metrics (DALYs, YLLs, YLDs, and mortality) attributable to the two malnutrition conditions across different SDI strata, genders and age subgroups. On this basis, decomposition estimation and health disparity analysis were conducted, followed by future burden forecasting. Key outcomes were summarized as below. First, overall age-standardized mortality and DALYs of wasting- and stunting-related diarrhea declined continuously from 1990 to 2021, with a more notable decreasing trend observed in girls. Second, along with the elevation of Socio-demographic Index (SDI), all burden indicators of nutrition-attributable diarrhea presented a steady downward trend. Third, the distribution characteristics and temporal trends of diarrhea burden related to wasting and stunting were generally consistent in different SDI regions, genders and age groups. Fourth, epidemiological improvement served as the core driving force for the global reduction in malnutrition-linked diarrhea burden. Fifth, the absolute health disparity of such diarrhea gradually narrowed, whereas relative inequality continued to rise.

Consistent with the overall trend, the age-standardized DALYs and mortality of malnutrition-attributable diarrhea declined steadily over the past three decades, with females experiencing a more obvious decline. In 2021, diarrhea deaths and mortality induced by wasting were higher than those caused by stunting. Globally, under-five boys exhibited higher mortality and death toll of nutrition-related diarrhea than girls, a common gender difference widely reported in epidemiological research. Multiple underlying mechanisms may explain this phenomenon. To begin with, male children are at higher risk of developing wasting and stunting (24). Moreover, biological variations in hormone secretion, immune competence and genetic regulation may also contribute to such disparity (25, 26). Genetically, females possess two X chromosomes, while males have only one; this enables females to obtain extra biological advantages through X-inactivation mediated chimerism (27). The unique genetic mechanism further endows females with more diversified protein expression and stronger physiological regulatory potential (28). Correspondingly, females demonstrate superior immune function, including enhanced antigen presentation, mitotic response of immune cells, and higher immunoglobulin levels (29). Accumulated evidence has also proven that male children are more susceptible to common infectious diseases (30). Furthermore, behavioral differences cannot be ignored: young boys are usually more active and curious about the external environment, which increases their exposure to various enteric pathogens. In practical public health work, targeted interventions should focus on male children, including daily hygiene management, safe drinking water guarantee, and standardized vaccination. For boys with diarrhea, medical staff and caregivers should attach sufficient clinical attention and provide timely oral rehydration to prevent dehydration and disease aggravation.

In 2021, an estimated 149 million under-five children worldwide suffered from stunting, and nearly 50 million suffered from wasting. The absolute number of age-standardized DALYs due to wasting-attributable diarrhea was lower than that of stunting, while its age-standardized DALY rate remained higher. It indicated that wasting was more closely correlated with severe diarrheal burden at the individual level. Nevertheless, due to the huge affected population of stunted children, stunting ultimately contributed to a higher overall diarrheal burden worldwide. Previous studies have demonstrated that stunting can be regarded as a chronic adaptive outcome of recurrent wasting, representing an adverse physiological adaptation to long-term severe malnutrition (24). Hence, early targeted intervention for wasted children is urgently needed. Such measures can not only reduce the acute diarrheal burden induced by wasting, but also block the irreversible progression from transient wasting to chronic stunting.

From 1990 to 2021, all burden indicators of malnutrition-related diarrhea decreased in tandem with increased SDI. The most remarkable burden reduction was detected in middle-high SDI regions. Low SDI and high SDI areas showed the smallest decline in DALY rates for wasting-attributable diarrhea, and low SDI regions had the weakest reduction in stunting-related diarrhea burden. Despite the global downward trend, low SDI regions remained the high-burden hotspots, concentrating the majority of wasted and stunted children. These underdeveloped areas face severe challenges including food shortage, inadequate medical infrastructure, poor sanitation, insufficient health education and unsafe drinking water (31). Global cooperation and multi-resource integration are urgently required to address these regional public health challenges.

From 1990 to 2021, the age-standardized mortality rate of diarrheal diseases attributable to wasting and stunting declined among children under five worldwide. This favorable trend was primarily attributed to global humanitarian interventions, including access to safe drinking water, improved sanitation facilities, rotavirus vaccination and oral rehydration therapy (ORT) (11, 32). Nevertheless, certain countries such as Sweden, Italy, Canada and Greece witnessed an upward trend in relevant mortality (33, 34) which was driven by multiple mechanisms involving shifts in enteric pathogen spectrum, growing proportion of vulnerable children and inappropriate feeding practices. The dominant cause of childhood diarrhea deaths in high-income countries has transitioned from rotavirus infection to mixed infections of various viruses, bacteria and parasites, with norovirus posing the most rapidly rising fatality risk. Reduced rotavirus-associated mortality has been fully offset and even surpassed by increasing deaths caused by other pathogens (35, 36). Although survival rates of very low birth weight infants (<1,500 g) and preterm infants have improved greatly in these regions, immature intestinal barrier and immune function render them highly susceptible to severe diarrhea, with their mortality 4–6 times higher than that of full-term infants (5). Over the past three decades, exclusive breastfeeding rates have dropped markedly among working mothers in Sweden, Canada and Italy. Infants without exclusive breastfeeding suffer impaired intestinal immunity and weakened defense against enteric pathogens, accompanied by elevated risks of severe diarrhea and death. A mild rise in childhood wasting further compromises physical resistance and exacerbates fatal outcomes of diarrheal diseases (34). It is necessary to explore country-specific influencing factors and formulate precise control measures.

Decomposition analysis confirmed that epidemiological progress was the primary factor driving the reduction in global malnutrition-related diarrhea burden. In contrast, continuous population growth exerted a counteractive effect and aggravated local disease pressure, especially in low SDI regions, where population expansion almost offset all health gains from epidemiological improvement. The global population is predicted to peak in the mid-2080s, rising from 8.2 billion in 2024 to around 10.3 billion. Rapid population growth will be mainly concentrated in low- and middle-income countries, especially sub-Saharan Africa and South Asia, which will further strain local diarrhea prevention systems (37). These regions are already trapped in structural disadvantages such as poor sanitation, limited medical accessibility and high malnutrition prevalence, which will be further aggravated by demographic changes (38). Major epidemiological advances include expanded rotavirus immunization, universal sanitation construction and standardized ORT promotion. Young children are highly vulnerable to rotavirus infection in early childhood. Before the widespread use of vaccines, rotavirus-induced diarrhea had long imposed a heavy health toll globally, and vaccination has effectively relieved such disease pressure (39). Even so, global health equity remains insufficient. By the end of 2020, nine countries in sub-Saharan Africa had not yet incorporated rotavirus vaccines into routine immunization programs. Continuous efforts are needed to optimize vaccine technology and expand affordable vaccination coverage in low-income regions (40).

In terms of health equity, during 1990–2021, the absolute health gap of malnutrition-attributable diarrhea between socioeconomically disadvantaged and advantaged groups narrowed, whereas relative health inequality gradually widened. The reduction in absolute disparity could be attributed to inclusive public health policies, poverty alleviation, socioeconomic development and improved social security systems. Even so, socioeconomic gaps still persist, and vulnerable populations continue to bear a heavier disease burden. Privileged groups can access high-quality medical resources and cutting-edge health services, leading to faster health improvement. Imbalanced resource allocation, inconsistent health policy implementation and unequal access to preventive services across different SDI settings hinder the balanced realization of global health goals (38).

ARIMA model predictions revealed a continuous annual decline in deaths and DALYs of diarrheal diseases caused by wasting and stunting from 2022 to 2050, with females experiencing a greater reduction in disease burden than males (25–29). Notably, the burden of wasting-attributable diarrhea decreased more significantly than that induced by stunting. This gender disparity may stem from females' inherent biological advantages and superior immune function, allowing them to derive greater health benefits from current prevention and treatment interventions. The more pronounced reduction in wasting-related burden also highlights that early nutritional interventions targeting wasted children can yield more effective long-term disease control outcomes. However, low-SDI regions remain hampered by rapid population growth and inadequate public health resources, which may compromise the anticipated burden reduction effects. Targeted regional support and gender-specific health strategies are crucial to narrow health gaps and accelerate the reduction of childhood malnutrition-related diarrheal burden by 2050.

## Conclusion

Over the past three decades, the global burden of childhood diarrhea attributable to wasting and stunting has declined steadily, accompanied by marked regional disparities in public health outcomes. Epidemiological advancements have contributed substantially to the global reduction in nutrition-related diarrheal burden, while population growth has intensified regional disease burden, nearly offsetting public health gains in low-SDI regions. Global absolute health inequalities associated with this condition have gradually narrowed, yet relative health inequities have continued to expand, with persistent regional and socioeconomic disparities undermining the effectiveness of unified global intervention policies. Notably, ARIMA model projections to 2050 indicate that wasting-related diarrheal burden will decline significantly more rapidly and profoundly than stunting-related burden, demonstrating distinct long-term improvement trajectories between the two nutritional risk factors. These results emphasize the need for stratified, targeted, and context-specific prevention and control strategies. Early nutritional interventions targeting children with wasting are essential to reduce acute diarrheal burden, whereas long-term growth surveillance and standardized nutritional management for stunted children can effectively mitigate associated chronic health impairments. Scaling up core public health initiatives, including safe water access, sanitation improvement, rotavirus vaccination, and standardized oral rehydration therapy, alongside the implementation of dynamic population and disease burden surveillance systems, is critical to address growing health inequities in high-burden low-SDI regions. Future research should investigate population-specific, regional, and phenotype-based mechanistic differences to optimize integrated prevention frameworks and promote equitable and sustainable global child health development.

### Study limitations

There exist several limitations in our research. First, estimates of diarrheal disease burden attributable to childhood stunting and wasting largely rely on the accessibility and quality of the GBD 2021 database. Some nations, particularly low- and middle-income countries, may have insufficient primary data, which can impede the estimation process among GBD researchers (22, 41, 42). Second, The GBD 2021 database relies on model-derived estimates instead of actual observational data, which may result in either overestimation or underestimation of the disease burden (22, 41, 42). Third, ARIMA projections are contingent on data quality and historical trends (43, 44). Childhood diarrhea is affected by a variety of drivers like climatic shifts and migratory patterns, and such models might incompletely reflect future developments, thereby introducing variability into the projections (45). Negative ARIMA projections lack concrete epidemiological meaning and should be interpreted only as relative trends for directional assessment.

## Data Availability

The original contributions presented in the study are included in the article/Supplementary Material, further inquiries can be directed to the corresponding author.
